# Cross-National Pain And Functional Impairment In Older Adults With CVD: Insights From India, China, And Mexico

**DOI:** 10.1093/geroni/igaf122.258

**Published:** 2025-12-31

**Authors:** Lu Shao, Hongtao Cheng, Huijun Wu, Junwen Yu, Jun-e Zhang

**Affiliations:** University of Hong Kong, Guangzhou, Guangdong, China (People’s Republic); Sun Yat-sen University, Guangzhou, Guangdong, China (People’s Republic); Sun Yat-sen University, Guangzhou, Guangdong, China (People’s Republic); Fudan University, Shanghai, Shanghai, China (People’s Republic); Sun Yat-sen University, Guangzhou, Guangdong, China (People’s Republic)

## Abstract

Chronic pain in older adults with cardiovascular disease (CVD) may exacerbate functional disability, yet cross-cultural evidence remains limited. This study examines pain-disability associations across India, China, and Mexico, providing a comparative perspective on aging and disability. We analyzed nationally representative data from three cohorts: the Longitudinal Aging Study in India (LASI, n = 11,247), China Health and Retirement Longitudinal Study (CHARLS, n = 3,733), and Mexican Health and Aging Study (MHAS, n = 5,108). Participants aged ≥60 with self-reported CVD were included. Chronic pain was assessed via self-report, and functional disability was measured using activities of daily living (ADL), instrumental ADL (IADL), and mobility scales. Cross-sectional multivariable logistic regression models examined associations, while longitudinal analyses in China and Mexico assessed 3-year incident disability risks. Pain prevalence was 43.7% in India, 36.2% in China, and 43.6% in Mexico. Adjusted models revealed significant associations between pain and ADL disability across all countries: India (OR = 1.86, 95% CI:1.70–2.04), China (OR = 2.22, 1.86–2.63), Mexico (OR = 2.32, 2.00–2.70). Pain severity showed dose-response relationships with ADL/IADL limitations. Longitudinally, baseline pain predicted higher risks of new-onset ADL disability in China (OR = 2.27, 1.71–3.01) and Mexico (OR = 1.97, 1.57–2.47), with consistent patterns for IADL disability. Chronic pain is a strong, independent predictor of functional disability in older CVD patients across diverse middle-income countries. These findings highlight the necessity of integrating pain management into cardiovascular care to promote functional independence in aging populations. Future research should explore culturally tailored interventions to mitigate the impact of pain on disability in diverse healthcare systems.

